# Teriparatide and Osteosarcoma Risk: History, Science, Elimination of Boxed Warning, and Other Label Updates

**DOI:** 10.1002/jbm4.10665

**Published:** 2022-08-14

**Authors:** John H Krege, Alicia W Gilsenan, John L Komacko, Nicole Kellier‐Steele

**Affiliations:** ^1^ Lilly Research Laboratories Eli Lilly and Company Indianapolis IN USA; ^2^ Department of Epidemiology RTI Health Solutions Research Triangle Park NC USA

**Keywords:** ANABOLICS, FRACTURE PREVENTION, OSTEOPOROSIS, PRIMARY TUMORS OF BONE AND CARTILAGE

## Abstract

The phase 3 trials of the bone anabolic drug teriparatide were prematurely terminated because of a preclinical finding of osteosarcoma in rats treated with high doses of teriparatide for near lifetime. Even so, results from these and subsequent clinical trials showed efficacy and tolerability. Based on the phase 3 results and additional preclinical investigations, Forteo (teriparatide) was approved for use in the United States with an indication for the treatment of osteoporosis in patients at high risk for fracture, a boxed warning regarding potential risk of osteosarcoma, a 2‐year lifetime limitation of use, other risk mitigations, and a requirement to assess for risk of osteosarcoma in humans treated with teriparatide. Subsequent investigations included five real‐world studies directed at assessing a connection between teriparatide and osteosarcoma risk in humans. The early studies did not identify an increased risk of osteosarcoma but were inadequate to sufficiently characterize risk, given the low incidence of this rare form of bone cancer. Learning from these efforts, two studies were undertaken using claims data to identify large cohorts of patients treated with teriparatide and assess whether these patients were found to have osteosarcoma by linking pharmacy claims data with data from cancer registries. These studies showed no increase in osteosarcoma in patients using teriparatide compared with unexposed groups, as well as to the expected population‐based background incidence of the disease. Based on this real‐world evidence and the totality of data collected from postmarketing use and other clinical investigations, the label was updated in 2020. The changes included addition of information from large observational studies using real‐world evidence, removal of the boxed warning, and a revision of the 2‐year lifetime limitation. Thus, observational studies with large sample sizes using real‐world data can provide supportive evidence to facilitate regulatory decisions including the elimination of a boxed warning. © 2022 Eli Lilly and Company. *JBMR Plus* published by Wiley Periodicals LLC on behalf of American Society for Bone and Mineral Research.

## Introduction

Osteoporosis and osteoporotic fractures can result in significant morbidity, increased mortality, reduced quality of life, and health care costs.^(^ ^1^, ^2^, ^3^ ^)^ With aging of the population, approximately 500 million people worldwide have been diagnosed with osteoporosis and the annual incidence of osteoporotic fractures is more than 8.9 million.^(^ ^4^, ^5^ ^)^

Teriparatide (Forteo, 20 mcg/d by subcutaneous injection, Eli Lilly and Company, Indianapolis, IN, USA) is a parathyroid hormone (PTH) analog with sequence identical to the first 34 amino acids of the 84‐amino acid human parathyroid hormone. Forteo was approved in the US in 2002 for the treatment of osteoporosis in patients at high risk of fracture. Findings from a lifetime carcinogenicity study in which teriparatide caused an increased incidence of osteosarcoma (OS) in rats resulted in the United States label required to include a boxed warning, and treatment duration was limited to 2 years.^(^ ^6^, ^7^ ^)^

This review focuses on the history of teriparatide development, identification of the potential osteosarcoma risk based on preclinical findings, studies directed at understanding whether teriparatide administration was associated with osteosarcoma risk in humans, recent updates to the teriparatide US prescribing information, and the clinical implications of those changes.

## Rationale for Development of Teriparatide

Preclinical studies demonstrated the potential of intermittent administration of PTH as an anabolic agent that stimulates osteoblasts and bone remodeling.^(^ ^8^, ^9^ ^)^ It was hypothesized that patients with osteoporotic fractures would benefit from an anabolic approach using intermittent PTH that would quickly rebuild the skeleton, although the anabolic therapy fluoride had failed to reduce fracture risk,^(^ ^10^ ^)^ and there was scientific uncertainty about the effects of teriparatide at cortical bone sites.^(^ ^11^ ^)^ Clinical development of daily subcutaneous teriparatide and a 2‐year preclinical toxicology study in rodents were undertaken.^(^ ^6^ ^)^

## Phase 3 Clinical Studies and Their Early Termination—Osteosarcoma in Preclinical Studies

A pivotal, phase 3, multinational, randomized, double‐blind, 3‐year clinical study was conducted to assess the efficacy of teriparatide in 1637 postmenopausal women with prior vertebral fracture.^(^ ^12^ ^)^ However, this study was prematurely terminated after a median of 19 months of treatment when the long‐term toxicology study in rats showed evidence of osteosarcoma after near lifetime exposure to teriparatide.^(^ ^6^ ^)^ Even so, when the clinical trial data from this study was initially and subsequently evaluated, results showed reductions in vertebral fractures, nonvertebral fractures, back pain, and increased bone mineral density.^(^ ^12^, ^13^, ^14^, ^15^ ^)^ A paired iliac crest bone biopsy study from a subset of patients showed an increase in trabecular bone volume and cortical thickness, and improvements in trabecular connectivity and structural model index.^(^ ^16^ ^)^ Post hoc analyses suggest teriparatide may be effective and safe in patients with renal insufficiency; data are limited in patients with severe renal impairment.^(^ ^17^, ^18^, ^19^ ^)^ Additional studies supported the findings that teriparatide was associated with clinical efficacy in patients with osteoporosis at high risk for fracture.^(^ ^20^, ^21^, ^22^, ^23^, ^24^, ^25^, ^26^ ^)^ Teriparatide had a novel mechanism of action compared with antiresorptive drugs and greater fracture risk reduction in a head‐to‐head fracture trial compared with a bisphosphonate.^(^ ^27^, ^28^, ^29^, ^30^, ^31^ ^)^ Teriparatide was generally well tolerated.

### Preclinical findings

**Fig. 1:**
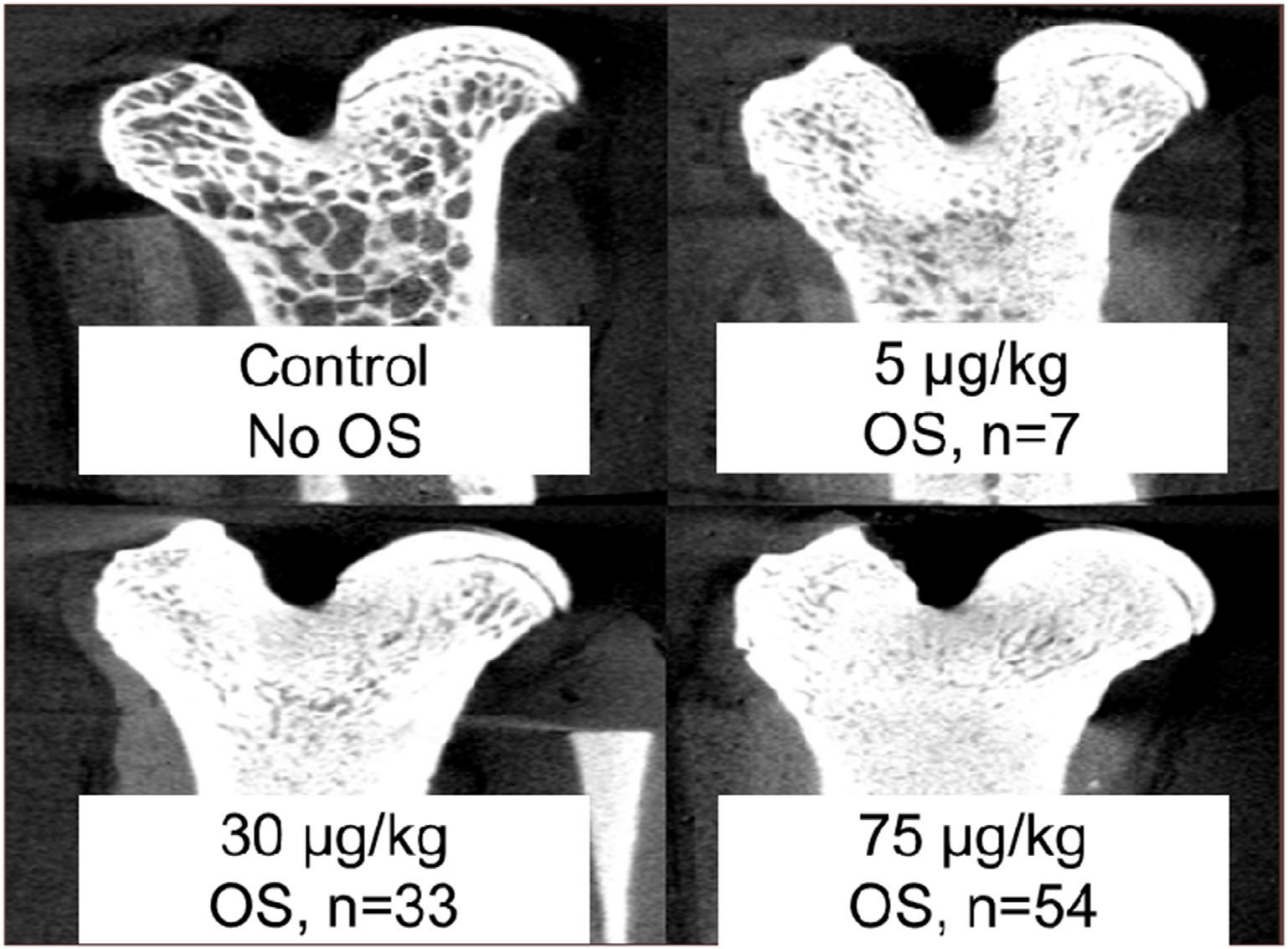
Skeletal changes and incidence of osteosarcoma in rats after exposure to different doses of once‐daily teriparatide versus control in first rodent toxicology study. The study included 60 animals per sex per group. OS = osteosarcoma.

Lilly performed one of the first long‐term toxicology studies of a recombinant biotherapeutic. Fisher rats (*n* = 344) were treated with once‐daily subcutaneous injections of control or supratherapeutic doses, including 5 μg/kg, 30 μg/kg, and 75 μg/kg teriparatide from ages 6 to 8 weeks.^(^ ^6^ ^)^ Results showed anabolism in rodent bone, with quantitative computed tomography (QCT) images showing excessive increases in bone mass with reduction and loss of the marrow space; the bone mineral content at the end of treatment was similar to that of a rod of pure bovine cortical bone. Osteosarcoma was observed in a dose‐dependent manner (Fig. 1), and daily hormonal stimulation of osteogenesis provided a biological rationale for the findings.

A second rodent toxicology study was designed. This study showed that treatment for 20 months at a clinically relevant dose did not result in bone tumors in the rat, establishing that teriparatide‐induced osteosarcomas in rats were both time‐ and dose‐dependent. Also, a study of ovariectomized monkeys treated with a teriparatide dose achieving 8 times the clinical exposure for 18 months and observation for 3 additional years showed no bone tumors.^(^ ^32^, ^33^ ^)^

Tashijian and colleagues reviewed the differences in bone physiology between rat and primates.^(^ ^34^ ^)^ Rats have a continuous, near‐lifetime skeletal growth and lack osteonal remodeling, whereas primate bones do not have continuous growth and bones are renewed by remodeling. These differences between the species resulted in an excessive bone anabolic response in the rat compared with the responses that were observed in monkey and in osteoporosis patients.^(^ ^34^ ^)^

After the initial rat toxicology study, Lilly established an oncology advisory board of osteosarcoma experts to consider these preclinical findings. The board concluded that the rat osteosarcoma findings were unlikely to predict development of bone tumors in humans. This was based on the following considerations: lifetime duration of treatment in rats compared with relatively brief exposure in humans, treatment was initiated in rats during relatively rapid growth phase, differences in rat and human bone biology and response to teriparatide, the finding of no bone tumors in the monkey study, and lack of association between hyperparathyroidism and osteosarcoma. No cases of osteosarcoma were observed in the clinical trials of patients treated with teriparatide. Based on the phase 3 studies demonstrating clinical efficacy and the evidence suggesting that the rodent findings were unlikely to be predictive of similar effects in humans, Lilly submitted a new drug application to the US FDA for use of teriparatide for the treatment of patients with osteoporosis.

## US FDA Approval of Teriparatide With a Boxed Warning, 2‐Year Limitation of Use, Risk Evaluation and Mitigation Strategies, and Postmarketing Requirements

The US FDA reviewed the teriparatide New Drug Application and convened an advisory board at which there was discussion of the benefits of teriparatide versus risks related to the preclinical toxicology findings.^(^ ^35^ ^)^ The advisory committee viewed the efficacy of teriparatide favorably but was not convinced that the safety profile had been fully defined based on the limited power of the clinical trials to detect an increase in osteosarcoma risk.^(^ ^36^ ^)^ Based on the medical need for an anabolic therapy and that the human risk of osteosarcoma based on the rodent toxicology studies was theoretical, the FDA approved Forteo (teriparatide) for the treatment of patients with osteoporosis at high risk of fracture to ensure patients had a sufficiently high medical need for anabolic therapy to outweigh the potential risks.^(^ ^37^ ^)^ Additional risk mitigation strategies including adequate labeling and inclusion of a Medication Guide were put in place. Labeling of the product included a boxed warning regarding the potential risk of osteosarcoma along with a recommendation to avoid the drug in patients at high risk for osteosarcoma. Labeling also stated that use of the drug for more than 2 years over a lifetime was not recommended. Additional risk mitigation measures included a Risk Evaluation and Mitigation Strategy with an education component to ensure that patients and prescribers were aware of the safety concerns related to the preclinical toxicology findings. Additionally, a postmarketing surveillance program was required to gain further insights into the potential risk of osteosarcoma in humans.^(^ ^37^ ^)^

## Postmarketing Surveillance Studies: Incidence of Osteosarcoma

A comprehensive program consisting of five post‐approval observational studies developed over more than 15 years was undertaken to characterize the risk of osteosarcoma among patients treated with teriparatide (Table 1). This work was iterative, with learning from each of the studies along the way guiding later studies.

**Table 1:** Overview of Study Design and Results of Postmarketing Studies for Teriparatide

	Osteosarcoma Surveillance Study^(^ ^7^ ^)^	European Osteosarcoma Surveillance Study^(^ ^36^ ^)^	Forteo Patient Registry^(^ ^37^ ^)^	Forteo Medicare Linkage Study^(^ ^38^ ^)^	Forteo Commercial Claims Linkage Study
Study design	Case series	Case series	Voluntary prospective cohort	Population‐based comparative cohort	Population‐based comparative cohort
Study location	USA	5 Nordic countries (Denmark, Finland, Iceland, Norway, Sweden)	USA	USA	USA
Outcome data source	Cancer registries^a^	Cancer registries	Cancer registries^a^	Cancer registries	Cancer registries
Exposure data source	Patient self‐report	Chart review	Patient self‐report	Medicare Part D prescription data	Commercial pharmacy claims data
Diagnosis years included	2003–2016	2004–2011	2009–2019	2007–2014	2005–2014
Ages included	≥40 years	≥40 years	≥18 years	≥65 years	≥18 years
Cancer Registry participation	30 cancer registries	Scandinavian Sarcoma Group (SSG) Registry and the Finnish and Swedish National Cancer Registries	42 cancer registries	26 cancer registries	29 cancer registries
No. of patients	1173 interviewed	109	6180 OS cases linked with 75,247 enrolled patients in Forteo Patient Registry	811 OS pts linked to 153,316 teriparatide and 613,247 comparators	4242 OS patients linked to: (1) 335,191 teriparatide and 637,387 osteoporosis and (2) 379,283 teriparatide and 1,428,943 general population
Results	3 patients with confirmed exposure to teriparatide before OS diagnosis	0 OS cases had a record of teriparatide use	0 teripartide users were found to have osteosarcoma	0 matches in teriparatide cohort; <11 matches in comparator cohort^b^	(1) Osteoporosis cohort: 3 matches in teriparatide and 6 in osteoporosis (2) General population cohort: 3 matches (same as in osteoporosis cohort) in teriparatide and 9 in general population
Statistical findings	SIR^c^: 0.72 (90% CI, 0.20–1.86)	IRR 0 (90% CI, 0‐27)^d^	Crude incidence rate: 0.0 (95% CI, 0.0–10.2) cases per million person‐years^d^	IRR: 0.0 (95% CI, 0.0–3.2)^d^	Osteoporosis IRR: 1.0 (95% CI, 0.2–4.5) general population IRR: 1.3 (95% CI, 0.2–5.1)^e^

CI = confidence interval; IRR = incident rate ratio; OS = osteosarcoma; SEER = Surveillance, Epidemiology, and End Results Program; SIR = standardized incidence ratio.

^a^ Responses were validated by medical record review in a random sample with >85% agreement.

^b^ The study comparator cohort included fewer than 11 observed cases. As a condition of the Medicare data use agreement, to protect patient privacy, nonzero cell counts less than 11 cannot be disclosed; thus, the exact number of cases cannot be reported because it is more than 0 but less than 11.

^c^ A background incidence rate of osteosarcoma of 3.2 cases per million per year was age‐ and sex‐adjusted to the teriparatide‐treated population and study interview rate was estimated. Based on these parameters, the expected number of cases of osteosarcoma with teriparatide exposure was 4.17; with three observed cases, the SIR was 0.72 (90% CI, 0.20–1.86).^(^ ^7^ ^)^

^d^ Because these studies showed no cases of osteosarcoma with teriparatide exposure, the incidence rate ratio was 0.

^e^ Because the observed number of cases with teriparatide exposure was similar to the expected number of cases in the osteoporosis and general population patients, the point estimates for the IRR were 1 and 1.3, respectively, indicating no observed increase in risk.

The first was the Osteosarcoma Surveillance Study, a 15‐year study initiated in 2003 as a postmarketing commitment to the US FDA. The study design and findings from the first 7 years were published in 2012^(^ ^38^ ^)^ and the final results in 2020.^(^ ^7^ ^)^ In this study, a case series design identified incident cases of osteosarcoma through cancer registries and used interviews to collect data regarding any exposure to teriparatide. Given the background rate of 3.2 cases per million per year (derived from NCI‐SEER rate), age‐ and sex‐adjusted to the teriparatide‐treated population, the expected number of cases was 4.17 cases. The study observed 3 cases and an estimated standardized incidence ratio of 0.72 (90% confidence interval [CI], 0.20–1.86).^(^ ^7^ ^)^ The results showed that osteosarcoma incidence with teriparatide use was not higher than the background incidence rate. In addition, another osteosarcoma surveillance study was conducted in five Nordic countries using regional and national cancer registry data to identify osteosarcoma cases and chart review to ascertain exposure to teriparatide. Among 109 cases of osteosarcoma identified, none had a record of teriparatide use. Given the limited number of osteosarcoma cases expected within the Nordic country population, the authors note that exposure to teriparatide among patients diagnosed with osteosarcoma would only be expected if there had been a large increased risk with the use of teriparatide.^(^ ^39^, ^40^ ^)^ Limitations for each of the studies included the number of osteosarcoma cases observed given this is a rare disease, the lack of a comparison group, and inability to control for potential confounders.

When teriparatide was approved for treatment of glucocorticoid‐induced osteoporosis, a new study termed the Forteo Patient Registry was added to the program in 2009. In the Forteo Patient Registry, patients receiving a Forteo prescription were requested to voluntarily register. Patients treated with Forteo were followed by linking their data to cancer registry data to identify patients developing osteosarcoma. In this study, 6180 osteosarcoma cases were linked with 75,247 patients in the Forteo Patient Registry, and no matches were found. Limitations of this study were that the study was not comparative and the number of patients enrolled was lower than the target number to evaluate the risk of developing osteosarcoma.^(^ ^41^ ^)^

Although these earlier studies did not suggest an increased risk of osteosarcoma in patients treated with teriparatide, they were inadequate to sufficiently characterize osteosarcoma risk. Accordingly, two large, population‐based, observational cohort studies were undertaken to identify a larger sample of patients treated with teriparatide using Medicare and a large commercial claims database. These pharmacy claims databases were linked to cancer registry data to identify cases of osteosarcoma among teriparatide‐treated patients. Teriparatide exposure was observed for 3 and 0 osteosarcoma cases among 379,283 and 153,316 teriparatide users, respectively.^(^ ^19^, ^40^, ^42^ ^)^ The study results suggested a similar risk for osteosarcoma between teriparatide users and their unexposed comparators.^(^ ^19^, ^40^, ^42^ ^)^

Overall, the real‐world data collected since 2003 through 2019 showed that the incidence rate of osteosarcoma in patients treated with teriparatide (2.47 million patients) did not exceed the background incidence rate.^(^ ^7^, ^40^, ^41^, ^42^, ^43^ ^)^ Additionally, observational studies of teriparatide use (without a focus on osteosarcoma) conducted in various geographies have also not reported any cases of osteosarcoma.^(^ ^44^, ^45^, ^46^, ^47^, ^48^ ^)^ Thus, an increased risk of osteosarcoma has not been observed in observational studies in humans.

## Osteosarcoma‐Related Updates in the Forteo (Teriparatide) US Prescribing Information

Based on the totality of data from the postmarketing surveillance studies (Table 1), other observational studies, postmarketing pharmacovigilance, and literature review, the FDA approved several updates to the Forteo (teriparatide) US prescribing information, which came into effect on November 16, 2020^(^ ^19^ ^)^ (Table 2). The findings from the Forteo Medicare linkage study and Forteo commercial claims linkage study showing a similar risk for osteosarcoma with teriparatide for 2 years or less and comparators were added to the label with a limitation regarding incomplete control for confounders (Tables 1 and 2).The boxed warning regarding the preclinical finding of osteosarcoma in rats was removed, although the warning/precaution describing the possible risk of osteosarcoma in humans was retained because of the rare frequency of the event, incomplete information regarding confounders, and limited information regarding use of teriparatide for more than 2 years.

**Table 2:** Important OS‐Related Updates in Forteo US Prescribing Information

Section	Previous version	Updated version^(^ ^40^ ^)^ (effective November 2020)	Supporting evidence
Boxed warning	In male and female rats, teriparatide caused an increase in the incidence of osteosarcoma (a malignant bone tumor) that was dependent on dose and treatment duration. The effect was observed at systemic exposures to teriparatide ranging from 3 to 60 times the exposure in humans given a 20‐mcg dose. Because of the uncertain relevance of the rat osteosarcoma finding to humans, prescribe Forteo only for patients for whom the potential benefits are considered to outweigh the potential risk. Forteo should not be prescribed for patients who are at increased baseline risk for osteosarcoma (including those with Paget's disease of bone or unexplained elevations of alkaline phosphatase, pediatric and young adult patients with open epiphyses, or prior external beam or implant radiation therapy involving the skeleton)	Boxed warning removed	Review of literature and findings from clinical trials, observational studies, pharmacovigilance and the postmarketing surveillance program did not show an increase in risk of OS in humans^(^ ^7^, ^38^, ^39^, ^41^, ^42^ ^)^
Warnings and precautions (osteosarcoma)	In male and female rats, teriparatide caused an increase in the incidence of OS (a malignant bone tumor) that was dependent on dose and treatment duration. Teriparatide should not be prescribed for patients with increased baseline risk of OS. These include: Paget's disease of bone. Unexplained elevations of alkaline phosphatase may indicate Paget's disease of bone pediatric and young adult patients with open epiphyses prior external beam or implant radiation therapy involving the skeleton	An increase in the incidence of OS (a malignant bone tumor) was observed in male and female rats treated with teriparatide. OS has been reported in patients treated with teriparatide in the postmarketing setting; however, an increased risk of OS has not been observed in observational studies in humans. There are limited data assessing the risk of OS beyond 2 years of teriparatide use. Avoid teriparatide use in patients at increased baseline risk of OS, including: open epiphyses (pediatric and young adult patients; teriparatide is not approved in pediatric patients) metabolic bone diseases other than osteoporosis, including Paget's disease of the bone bone metastases or a history of skeletal malignancies prior external beam or implant radiation therapy involving the skeleton hereditary disorders predisposing to OS	Results of the postmarketing surveillance program did not show an increase in risk of OS in humans^(^ ^7^, ^38^, ^39^, ^41^, ^42^ ^)^
Dosage and administration: treatment duration	Use of the drug for more than 2 years during a patient's lifetime is not recommended.	Use of teriparatide for more than 2 years during a patient's lifetime should only be considered if a patient remains at or has returned to having a high risk for fracture.	Review of literature and findings from clinical trials, observational studies, and the postmarketing surveillance program did not show an increased risk of osteosarcoma in humans treated with teriparatide. However, studies evaluating efficacy of teriparatide for >2 years are limited.^(^ ^47^, ^48^, ^49^, ^50^ ^)^
Adverse reactions	—	Added the results of postmarketing observational database studies.^(^ ^38^, ^39^, ^40^ ^)^ **Adverse reactions from observational studies to assess incidence of osteosarcoma** “Two postmarketing observational studies identified 3 and 0 OS cases among 379,283 and 153,316 teriparatide users, respectively, in two U.S. claims‐based databases. The study results suggest a similar risk for OS between teriparatide users and their comparators. However, the interpretation of the study results calls for caution owing to the limitations of the data sources (prescription data only) which do not allow to measure and control for confounders.”	The number of patients in these studies were higher compared with other postmarketing studies. These two observational studies showed a similar risk for OS with teriparatide use of 2 years or less and comparators.

AE = adverse events; OS = osteosarcoma.

Another change was the lifetime restriction on duration of use of teriparatide for up to 2 years was adjusted to allow patients remaining at or who have returned to having a high risk for fracture to take teriparatide beyond 2 years (Table 2).

## Clinical Implications of the Label Updates

The updates to the Forteo (teriparatide) label provide patients and clinicians with the information that an increased risk of osteosarcoma has not been observed in humans treated with teriparatide. The information from teriparatide clinical trials, observational studies, and the postmarketing surveillance program can help clinicians in benefit–risk assessments and making informed decisions on treatment strategy for patients with osteoporosis who are at high risk of fracture.

Some patients who complete 24 months of treatment with teriparatide remain at high risk for fracture or return to a state of high risk for fracture after a previously completed course. These patients may benefit from additional treatment or retreatment with teriparatide. Thus, modification of the 2‐year lifetime limitation of use may provide prescribers an opportunity to tailor the treatment duration and manage their patients based on their individual needs.

Recently, Miller and colleagues published some clinical situations in which treatment with teriparatide beyond 2 years may be beneficial.^(^ ^49^ ^)^ These included patients with osteoporosis and very high fracture risk and dependency on glucocorticoid therapy, high fracture risk and high P1NP levels after 2 years of treatment with teriparatide, high fracture risk with multiple vertebral compression fractures at baseline while none with teriparatide, adynamic renal bone disease, severe chronic obstructive pulmonary disease, and vertebral compression fractures.^(^ ^49^ ^)^

## Limitations and Future Directions

There are some limitations of the currently available data for teriparatide. The efficacy and safety data beyond 2 years of treatment with teriparatide is limited both in terms of continuous treatment as well as retreatment.^(^ ^50^, ^51^, ^52^, ^53^ ^)^ Data are not available to guide the duration of additional treatment beyond 2 years. Therefore, the risk of osteosarcoma in humans with longer duration of treatment is not fully understood, requiring physician judgment of benefit/risk in individual patients. Consequently, the updated label reflects that use of teriparatide for more than 2 years during a patient's lifetime should only be considered if a patient remains at or has returned to having a high risk for fracture. Observational studies using pharmacy claims data have some limitations. The limitations for the linkage studies using Medicare and commercial pharmacy claims include an inability to assess detailed baseline patient characteristics and medical histories (only prescription data were available in the study data sources) and these study designs could not completely measure and control for confounding. It is unknown whether findings from patients treated with teriparatide are relevant to other anabolic treatments, such as PTHrP analogs.

In summary, the initial phase 3 trials of the bone anabolic drug teriparatide were stopped because of a preclinical finding of osteosarcoma in rats treated with high doses of teriparatide for near lifetime. Even so, teriparatide was observed to reduce fracture risk in postmenopausal women with osteoporosis and showed efficacy and tolerability in the phase 3 program and in subsequent studies with a positive benefit to risk profile. Based on the phase 3 results, additional preclinical findings, and with risk mitigations, the agent was approved for use. Subsequent studies including in real‐world settings have not identified an increased risk of osteosarcoma in humans, and the label has been updated and adjusted, including removal of the boxed warning and a revision of the 2‐year lifetime limitation. Thus, observational studies with large sample sizes using real‐world data can be an important and efficient strategy for generating evidence in support of regulatory decision making and significant label updates.

## Disclosures

All authors have completed the ICMJE uniform disclosure form. JHK, JLK, and NKS are employees of Eli Lilly and Company. AG is an employee of RTI Health Solutions.

## Author Contributions

**John H Krege:** Conceptualization; writing – review and editing. **Alicia W. Gilsenan:** Data curation; formal analysis; investigation; methodology; writing – review and editing. **John L Komacko:** Writing – review and editing. **Nicole Kellier‐Steele:** Conceptualization; data curation; formal analysis; investigation; methodology; writing – review and editing.

## Ethical Statement

This manuscript describes retrospective data that does not require ethics committee approval.

## Data Availability

Data sharing not applicable to this article as no data sets were generated or analyzed during the current study.
